# Kosten-Erlös-Aspekte der endovaskulären Versorgung distaler Aortenbogenpathologien im Hinblick auf die Einführung einer neuen thorakalen Seitenarmprothese

**DOI:** 10.1007/s00104-024-02072-3

**Published:** 2024-03-18

**Authors:** Moritz S. Bischoff, Denis Skrypnik, Wolfgang Fiori, Oliver Schöffski, Dittmar Böckler

**Affiliations:** 1 Klinik für Gefäßchirurgie und Endovaskuläre Chirurgie, Universitätsklinikum Heidelberg, Im Neuenheimer Feld 420, 69120 Heidelberg, Deutschland; 2 DRG-Research-Group, Roeder & Partner Ärzte PartG, Senden, Deutschland Am Dorn 10, 48308; 3 Lehrstuhl für Gesundheitsmanagement, Universität Erlangen-Nürnberg, Nürnberg, Deutschland Lange Gasse 20, 90403

**Keywords:** Gefäßchirurgie, Aortenbogenpathologie, Hybridverfahren, TEVAR, Kosten-Nutzen-Analyse, Vascular surgery, Aortic arch pathologies, Hybrid approach, Thoracic endovascular aortic repair, Cost-revenue analysis

## Abstract

**Hintergrund:**

Das gefäßchirurgische Standardverfahren (SV) für die Versorgung distaler Aortenbogenpathologien ist ein Hybridengriff aus linksseitigem karotidosubklavialem Bypass und thorakaler endovaskulärer Aortenreparatur. Vor dem Hintergrund der Einführung einer thorakalen Seitenarmprothese (TBE) war das Ziel dieser Arbeit eine Analyse der Kosten-Erlös-Aspekte beider Verfahren.

**Material und Methoden:**

Es erfolgte eine retrospektive Aufarbeitung von Fällen, die 2017 bis 2022 mittels SV operiert wurden. Um Rückschlüsse auf den Einsatz der TBE vornehmen zu können, wurden für die Erlösberechnungen Hauptdiagnosen und Prozeduren des SV anhand aktueller Klassifikationen (ICD/OPS 2023) rekodiert und nach aG-DRG 2023 neugruppiert. Für die Modellierung der TBE-Erlöse erfolgte eine OPS-Änderung und Neugruppierung.

**Ergebnisse:**

Es konnten 13 Fälle identifiziert werden (mittleres Alter: 62,5 ± 13,8 Jahre; 10 Männer). Nach Neugruppierung lagen folgende DRGs vor: F42Z in *n* = 5, F51A in *n* = 4, F08B in *n* = 2 sowie F07A und F36B in je *n* = 1. Der Gesamterlös nach Neugruppierung betrug 666.514,13 €, inklusive Zusatzentgelt (ZE) von 132.729,14 €. Bei modellierter TBE-Anwendung wurde ein Gesamterlös von 659.212,19 € erzielt. Im Vergleich zum SV entspricht dies einem Erlösrückgang um 16.886,71 € (geänderte DRG), bei jedoch um 65.559,78 € steigenden ZE-Erlösen (unterschiedliche ZE). Bei TBE-Anwendung können 74 Belegungstage, davon 13,5 Intensivstationsbelegungstage, eingespart werden.

**Schlussfolgerung:**

Eine Kostenüberdeckung erscheint bei Verfahrenswechsel wahrscheinlich – trotz noch nicht geklärter Bepreisung der TBE. Dies ist, bei sich jährlich ändernden DRG-Relativgewichten, in hohem Maße abhängig von der Kodierqualität und der zukünftigen Entwicklung der ZE. Für deren Festsetzung wird eine präzise und transparente Leistungs- und Kostenerfassung benötigt.

**Zusatzmaterial online:**

Zusätzliche Informationen sind in der Online-Version dieses Artikels (10.1007/s00104-024-02072-3) enthalten.

## Abstract (en)

**Background:**

The standard vascular surgical procedure (SV) for the treatment of distal aortic arch pathologies involves a hybrid approach using a left carotid-subclavian bypass and thoracic endovascular aortic repair. Considering the introduction of a thoracic side branch prosthesis (TBE), the aim of this study was to analyze the cost-revenue aspects of both procedures.

**Material and methods:**

A retrospective analysis was conducted on cases treated by SV from 2017 to 2022. To draw conclusions regarding the use of TBE, the main diagnoses and procedures of SV were recoded based on current classifications (ICD/OPS 2023) for revenue calculations and regrouped according to aG-DRG 2023. An OPS modification and regrouping were performed for modeling TBE revenues.

**Results:**

A total of 13 cases were identified (mean age 62.5 ± 13.8 years; 10 males). After regrouping, the following DRGs were obtained: F42Z in *N* = 5, F51A in *N* = 4, F08B in *N* = 2, and F07A and F36B each in *N* = 1. The total revenue after regrouping was € 666,514.13, including an additional payment (ZE) of € 132,729.14. With the modeled application of TBE, a total revenue of € 659,212.19 was achieved. Compared to SV, this represents a revenue decrease of € 16,886.71 (changed DRG), but with an increase in ZE revenue by € 65,559.78 (different ZE). The use of TBE resulted in a saving of 74 occupancy days, including 13.5 days in intensive care.

**Conclusion:**

A cost coverage seems probable with a change in the procedure, despite the yet to be determined pricing of TBE. This is highly dependent on the coding quality and the future development of ZE, given the annually changing DRG relative weights. Precise and transparent performance and cost documentation are essential for determining the pricing.

## Hintergrund und Fragestellung

Die moderne Aortenchirurgie ist geprägt von den Verfahren der endovaskulären Therapie. Seit Beginn der 2000er-Jahre kam es hierbei zu einer stetigen Verbesserung auf allen drei entscheidenden Ebenen: der Patientenselektion, der intra- und perioperativen Expertise und Logistik sowie dem dabei eingesetzten Material, insbesondere bei den Stentgraft-Prothesen (SG).

(SG) sind im Rahmen der thorakalen endovaskulären Aortenreparatur (TEVAR) erheblichen Anforderungen hinsichtlich ihrer technischen Konstruktion in einem Flexibilitäts-Stabilitäts-Kontinuum unterworfen [1]. Über die Zeit wurden die Prothesen flexibler, um auch in anspruchsvollen Anatomien gut und sicher positioniert werden zu können. Die Einführbestecke wurden sukzessive in ihrem Durchmesser reduziert. Des Weiteren wurden zahlreiche Modifikationen der Freisetzungs- und Adaptationsmechanismen vorgenommen [2]. Allerdings haben die Fortschritte in der Medizintechnik in Kombination mit hohen Materialkosten auch zu relevanten Kostensteigerungen geführt.

Die Implementierung eines neuen Verfahrens in das operative gefäßchirurgische Portfolio kann aus gesundheitsökonomischer Sicht aufgrund begrenzter wirtschaftlicher Ressourcen nicht mehr nur nach rein klinischem Verständnis und Zielsetzung erfolgen. Vielmehr verlangen die hohen Materialkosten neuer Techniken sorgfältige Analysen hinsichtlich ihres medizinischen Mehrwerts und ihrer ökonomischen Effizienz gegenüber den bestehenden Therapieoptionen. Dieses sollte im Optimalfall bereits vor der Einführung eines neuen Verfahrens in den klinischen Alltag erfolgen. Für die endovaskuläre Aortenchirurgie stellt die bevorstehende Einführung einer neuen thorakalen SG (Thoracic Branch Endoprothese [TBE], W. L. Gore & Associates, Flagstaff, Arizona, USA) mit Seitenarm (für die linke Arteria subclavia, lSCL) eine wichtige Neuerung in der Versorgung von Aortenpathologien im distalen Aortenbogen dar (proximale Landungszone 2; [15]). Bis dato besteht das klinikinterne Standardverfahren für die Versorgung dieser Pathologien aus einem (zweizeitigen) Hybrideingriff, bestehend aus offen-chirurgischem Bypassverfahren für die lSCL (karotidosubklavialer Bypass [CSCL-Bypass]) und anschließender TEVAR [3, 5].

Das Ziel der vorliegenden Studie war daher eine Charakterisierung des bisherigen Hybrideingriffs bez. seiner Kosten-Erlös-Aspekte. Anhand der erhobenen Ergebnisse wurde dann die Einführung der neuen thorakalen Seitenarmprothese simuliert sowie im Anschluss diskutiert.

## Studiendesign und Untersuchungsmethoden

### Studiendesign

Bei der vorliegenden Studie wurde auf retrospektive Falldaten aus den Jahren 2017 bis 2022 zurückgegriffen. Es handelt sich daher auf Fallniveau um eine retrospektive, monozentrische Studie. Mit dieser historischen Kohorte wurden Erlös- und Kostenkalkulationen vor dem Hintergrund der bevorstehenden Einführung der TBE durchgeführt.

### Falldaten

Die zugrunde liegenden Falldaten entstammen einer prospektiv geführten TEVAR-Datenbank (Ethikvotum S‑185/2015) der Klinik für Gefäßchirurgie und Endovaskuläre Chirurgie des Universitätsklinikums Heidelberg (Ärztl. Direktor: Univ.-Prof. Dr. med. Dittmar Böckler).

### Ein- und Ausschlusskriterien

Die Ein- und Ausschlusskriterien sind in Tab. 1 dargestellt.

**Table 1:** 

	Einschlusskriterien	Ausschlusskriterien
Behandlungsmodalität	TEVAR mit PLZ 2	TEVAR außerhalb PLZ 2
Dringlichkeit	Elektiveingriffe	Notfälle/dringliche Eingriffe
Aortenpathologie	TAA, ADB, PAU	Andere Pathologien
Versorgung	1. CSCL-Bypasses 2. TEVAR in PLZ 2	Abweichendes Vorgehen zu Einschlusskriterien
Verwendete SG	N = 1	N ≠ 1
Mehrzeitigkeit	CSCL-Bypass vor TEVAR^a^	CSCL-Bypass simultan/nach TEVAR
Zeitpunkt	2017 bis 2022	Außerhalb 2017 bis 2022

*PLZ* proximale Landungszone, *TAA* thorakales Aortenaneurysma, *ADB* Aortendissektion Typ Stanford B, *PAU* penetrierendes Aortenulkus, *CSCL* karotidosubklavialer Bypass, *TEVAR* thorakale endvaskuläre Aortenreparatur, *=* gleich, *≠* ungleich

^a^ In einem stationären Aufenthalt an zwei unterschiedlichen Tagen

### Verwendete Definitionen und Klassifikationssysteme

Die Verschlüsselung von Diagnosen erfolgte nach der 10. Revision der Internationalen statistischen Klassifikation der Krankheiten und verwandter Gesundheitsprobleme, German Modification (ICD-10-GM, Stand: Januar 2023; [14]). Für die Kodierung von Operationen/Prozeduren wurde auf den Operationen- und Prozedurenschlüssel (OPS) 2023 zurückgegriffen [13]. Die Kodierung erfolgte unter Anwendung der Deutschen Kodierrichtlinien (DKR) 2023 [10]. Bezüglich der klinikinternen Implantationstechnik bei TEVAR und logistischer periprozeduraler Details wird auf die veröffentlichten Implantationsprotokolle verwiesen [8, 9].

### Datenextraktion und Aufbereitung der Falldaten

Unter Beachtung der Ein- und Ausschlusskriterien (Tab. 1) erfolgte die Datenabfrage aus der klinikinternen TEVAR-Datenbank (Microsoft EXCEL, Microsoft Corporation, Redmond, Washington, USA). Bei der erweiterten Datenerhebung kam die klinikinterne Datenverwaltungssoftware (i.s.h.med, SAP SE, Walldorf, Deutschland) zur Anwendung. Im Anschluss erfolgte anhand der jeweiligen individuellen Fallnummer in Zusammenarbeit mit dem Finanzcontrolling die Datenerhebung bzw. Datenzusammenführung auf Fallebene unter Berücksichtigung der Vorgaben des § 301 Sozialgesetzbuch V und des § 21 Krankenhausentgeltgesetz (KHEntgG). Die Verarbeitung der Daten erfolgte als Comma-separated values (.CSV). Um die Einzelfälle DRG-Fallpauschalen zuzuordnen, wurde ein vom InEK zertifizierter Grouper eingesetzt (GetDRG der Gesellschaft für den Einsatz offener Systeme mbH, GEOS).

## Berechnungen

### Verweildauerberechnung

Folgende fallbezogene Verweildauern wurden differenziert: Gesamtverweildauer (Entlassung – Aufnahme, abzüglich Tage ohne Berechnung und Intervallen von Fällen, bei denen es zu einer Fallzusammenführung gekommen ist), CSCL-Bypass – Aufnahme, TEVAR – CSCL-Bypass, Entlassung – TEVAR (jeweils in Belegungstagen). Zusätzlich dokumentiert wurden Gesamtintensivstationsverweildauer, Intensivstationsverweildauer vor CSCL-Bypass, zwischen CSCL-Bypass und TEVAR sowie nach TEVAR (jeweils in Tagen). Anhand dieser Aufschlüsselung waren die Auswirkungen eines Verfahrenswechsels hin zur TBE auf die Belegung von Normal- bzw. Intensivstation simulierbar.

### Erlösberechnungen

Grundlage der Erlösberechnungen war der vom Institut für das Entgeltsystem im Krankenhaus (InEK) publizierte Fallpauschalenkatalog bzw. Pflegeerlöskatalog 2023 [11]. Demnach erfolgten Gruppierung in Diagnosis Related Groups (DRG), Zuordnung von Zusatzentgelten (ZE) sowie des Pflegeerlöses nach der G‑DRG-Version 2023. Für die Kalkulation der Fallpauschalenerlöse erfolgte eine Multiplikation des Zahl-Landesbasisfallwerts Baden-Württemberg aus 2023 (4022,56 €) mit dem jeweiligen Relativgewicht der entsprechenden DRG (DRG-Erlös = Relativgewicht × Basisfallwert; [4]). Die Pflegeerlösberechnung erfolgte anhand der berechnungsrelevanten Verweildauertage (Belegungstage) mit dem für das Universitätsklinikum Heidelberg seit 01.03.2023 gültigen tagesbezogenen Pflegeentgeltwert (nach § 7 KHEntgG; 406,19 €) sowie der Bewertungsrelation (§ 17b Krankenhausfinanzierungsgesetz, KHG; Pflegeerlös = Pflegeerlösbewertungsrelation × Anzahl Verweildauertage × Pflegeentgeltwert). Für die Bewertung der ZE wurde auf im aG-DRG-Fallpauschalenkatalog 2023 differenzierte Beträge bzw. auf krankenhausindividuelle Entgelte (nach § 6 KHEntgG) zurückgegriffen (Stand: DRG-Entgelttarif des Universitätsklinikums Heidelberg vom 01.03.2023; [6, 11]). Tab. 2 zeigt die beim Standardverfahren bzw. TBE zu kodierenden OPS sowie die ausgelösten ZE [6].

**Table 2:** 

Standardverfahren	TBE
CSCL-Bypass: 5‑393.02	Prothese: 5‑38a.7b
TEVAR: 5‑38a.70	Seitenarm: 8‑842.08
VP: 8‑836.m8 (Lokalisation) OPS 8‑836.n1 (Anzahl) 8‑83b.34 (Material)	Bioaktive Oberfläche: 8‑83b.e1
Hybridverfahren: 5‑38a.a	–
ZE2023-50: 10.000 €	ZE2023-189: 15.249,75 €
ZE105.1: 206,69 €	–

*TBE* „thoracic branch endoprosthesis“, *CSCL* karotidosubklavialer Bypass, *TEVAR* thorakale endvaskuläre Aortenreparatur, *VP* Verschlussköper Arteria subclavia, *ZE* Zusatzentgelt (nach [6, 13])

Für die Erlössimulation bez. der TBE erfolgten folgende Schritte:

1. Die OPS 5‑393.02 (CSCL-Bypass) wurde durch die OPS der TBE ersetzt:
  5‑38a.7b: *Endovaskuläre Implantation von Stent-Prothesen: Ao. thoracica: Stent-Prothese, mit 1 Öffnung*
  8‑842.08: *(Perkutan-)transluminale Implantation von nicht medikamentenfreisetzenden gecoverten Stents (Stent-Graft): Ein Stent: Andere Gefäße thorakal*
  8‑83b.e1: *Zusatzinformationen zu Materialien: Art der Beschichtung von Stents: Bioaktive Oberfläche bei gecoverten Stents*
2. Die OPS 5‑38a.70 (TEVAR) und 5‑38a.a (Hybridverfahren) wurden gelöscht. Ebenso erfolgte eine Löschung des OPS für den Vascular Plug.
3. Die Verweildauer zwischen CSCL-Bypass und TEVAR wurde von der Gesamtverweildauer abgezogen (ganze Belegungstage).

### Kostenberechnungen

Die Berechnung der Kosten erfolgte anhand von Datensätzen, welche das Universitätsklinikum Heidelberg im Rahmen der jährlichen DRG-Fallkostenkalkulation erstellt hat. Die Kostenaufstellungen wurden in Zusammenarbeit mit dem Medizin- und Finanzcontrolling durchgeführt. Die Darstellung der Kosten erfolgte nach Vorgaben des InEK für die Methodik zur Kalkulation fallbezogener Behandlungskosten im Krankenhaus (sog. InEK-Matrix). Für die vorliegende Studie wurden die Kosten der untersuchten Fälle in dieser Matrix aggregiert (nach [12]).

## Statistik

Für die vorliegende explorative Studie wurden deskriptive statistische Verfahren verwendet. Numerische Variablen werden anhand des Mittelwertes und der Standardabweichung bzw. Spannweite beschrieben. Kategoriale Variablen werden durch Angabe der absoluten und relativen Häufigkeiten beschrieben. Alle Berechnungen wurden mittels Microsoft EXCEL (Version 16.71, Microsoft Corporation, Redmond, WA, USA) durchgeführt.

## Ergebnisse

### Fälle und zugehörige DRGs

Anhand der angelegten Ein- und Ausschlusskriterien konnten in den Jahren 2017 bis 2022 insgesamt 13 Fälle für die Analyse identifiziert werden. Der Frauenanteil betrug 3 von 13. Das mittlere Alter betrug 62,5 ± 13,8 Jahre (Spannweite: 41–84 Jahre). Im Jahr 2017 wurden zwei, im Jahr 2018 sechs, im Jahr 2019 drei und im Jahr 2022 zwei Fälle operiert.

Die aortale Aufnahme- bzw. Hauptdiagnose war in 7 von 13 Fällen eine thorakoabdominelle Aortendissektion (ADB; I71.03: *Dissektion der Aorta, thorakoabdominal, ohne Angabe einer Ruptur*). In 6 von 13 Fällen lag als aortale Hauptdiagnose ein penetrierendes Aortenulkus (PAU; I77.80: *Penetrierendes Aortenulkus)* vor.

Insgesamt wurden die 13 analysierten Fälle durch eine Grouper-Software in die fünf folgende DRGs gruppiert: F36B, F07A, F42Z, F08B und F51A. Tab. 3 fasst die wichtigsten Fallpauschalenmerkmale zusammen. Ausgehend von der DRG-Bewertungsrelation und ggf. dem Langliegerzuschlag (abhängig von der Verweildauer) ergibt sich durch Summe der DRG-Bewertungsrelationen der Case Mix als Ausgangsschritt für die Erlösberechnungen.

**Table 3:** 

Fall #	DRG	BWR	MVD (Tage)	Zuschlag ab (Tag)	VWD	LLZ	CM
1	F42Z	6,189	13,3	27	30	0,692	6,881
2	F51A	4,817	8,2	17	10	0	4,817
3	F42Z	6,189	13,3	27	13	0	6,189
4	F51A	4,817	8,2	17	15	0	4,817
5	F08B	5,315	21,3	39	21	0	5,315
6	F36B	10,619	26	44	44	0,264	10,883
7	F07A	6,75	14,1	28	38	3,058	9,808
8	F42Z	6,189	13,3	27	35	1,557	7,746
9	F42Z	6,189	13,3	27	13	0	6,189
10	F42Z	6,189	13,3	27	27	0,173	6,362
11	F08B	5,315	21,3	39	34	0	5,315
12	F51A	4,817	8,2	17	9	0	4,817
13	F51A	4,817	8,2	17	10	0	4,817
*Summe*	*–*	*–*	*–*	*–*	*–*	*–*	*83,956*

*#* Nummer, *DRG* Diagnosis Related Group, *BWR* Bewertungsrelation basierend auf dem aG-DRG-System 2023, *MVD* mittlere arithmetische Verweildauer basierend auf dem aG-DRG-Fallpauschalenkatalog [11], *VWD* Verweildauer, *LLZ* Langliegerzuschlag basierend auf dem aG-DRG-System 2023, *CM* Case Mix basierend auf dem aG-DRG-System 2023 (nach [10–12])

### Erlösberechnungen für das Standardverfahren

Die Erlösrechnung für das Standardverfahren im Detail zeigt Tab. 4. Es ergibt sich ein Gesamterlös von 666.514,13 €.

**Table 4:** 

Fall #	DRG	VWD	CM	Erlös BFW BW^1^	BWR FP	HOL-Zuschlag	BWR Pflege FP	CM Pflege	Pflegerlös^2^	ZE2023-50^3^	ZE105.01	ZE101.01	Summe Erlöse^4^
1	F42Z	30	6,881	27.679,24 €	6,189	0,692	1,4478	43,434	17.642,46 €	10.000,00 €	206,69 €	–	55.528,38 €
2	F51A	10	4,817	19.376,67 €	4,817	0	1,1676	11,676	4742,67 €	10.000,00 €	206,69 €	–	34.326,04 €
3	F42Z	13	6,189	24.895,62 €	6,189	0	1,4478	18,8214	7645,06 €	10.000,00 €	206,69 €	–	42.747,38 €
4	F51A	15	4,817	19.376,67 €	4,817	0	1,1676	17,514	7114,01 €	10.000,00 €	206,69 €	–	36.697,37 €
5	F08B	21	5,315	21.379,91 €	5,315	0	1,0308	21,6468	8792,71 €	10.000,00 €	206,69 €	–	40.379,31 €
6	F36B	44	10,883	43.777,52 €	10,619	0,264	2,7744	122,0736	49.585,08 €	10.000,00 €	206,69 €	–	103.569,29 €
7	F07A	38	9,808	39.453,27 €	6,75	3,058	2,1504	81,7152	33.191,90 €	10.000,00 €	206,69 €	–	82.851,86 €
8	F42Z	35	7,746	31.158,75 €	6,189	1,557	1,4478	50,673	20.582,87 €	10.000,00 €	206,69 €	–	61.948,31 €
9	F42Z	13	6,189	24.895,62 €	6,189	0	1,4478	18,8214	7645,06 €	10.000,00 €	206,69 €	–	42.747,38 €
10	F42Z	27	6,362	25.591,53 €	6,189	0,173	1,4478	39,0906	15.878,21 €	10.000,00 €	206,69 €	–	51.676,43 €
11	F08B	34	5,315	21.379,91 €	5,315	0	1,0308	35,0472	14.235,82 €	10.000,00 €	206,69 €	42,17 €	45.864,59 €
12	F51A	9	4,817	19.376,67 €	4,817	0	1,1676	10,5084	4268,41 €	10.000,00 €	206,69 €	–	33.851,77 €
13	F51A	10	4,817	19.376,67 €	4,817	0	1,1676	11,676	4742,67 €	10.000,00 €	206,69 €	–	34.326,04 €
*Summe*	*–*	*–*	*–*	*337.718,05* *€*	*–*	*–*	*–*	*–*	*196.066,94* *€*	*130.000,00* *€*	*2686,97* *€*	*42,17* *€*	*666.514,13* *€*

*#* Nummer, *VWD* Verweildauer, *BWR* Bewertungsrelation, *CM* Case Mix (Summe der Bewertungsrelationen), *BFW* Basisfallwert, *BW* Baden-Württemberg, *FP* Fallpauschale (DRG), *HOL* High Outlier (Langlieger), *ZE* Zusatzentgelt

^1^ Zahl-Landesbasisfallwert BW 2023: 4022,56 €

^2^ Pflegeentgeltwert aus aktuellem Entgelttarif vom 1. März 2023: 406,19 €

^3^ Wert aus Entgelttarif gültig ab 1. März 2023

^4^ Ohne weitere Zu- und Abschläge; basierend auf dem aG-DRG-System 2023. Das ZE101.01 (medikamentenfreisetzender Koronarstent) wurde in einem Einzelfall aufgrund einer Komplikation erlöst

### Kosten- und Verweildauerberechnungen für das Standardverfahren

Die gemittelten Fallkosten der 13 untersuchten Fälle sind als Zusatzmaterial (Supplement Tab. 1) verfügbar. Die Gesamtkosten aller Fälle betrugen 469.437,65 €, wobei dem Operationsbereich mit 217.427,24 € der größte Anteil zufiel. Der Fokus der Kostendarstellung liegt hier insbesondere auf den Implantatkosten (v. a. verwendete SG), welche der Einzelkostenzuordnung unterfallen. Die Verweildaueraufstellung ist ebenfalls als Zusatzmaterial verfügbar (Supplement Tab. 2).

### Erlössimulation für die TBE

Anhand der in der Methodik geschilderten Vorgehensweise erfolgte die Erlössimulation für den Einsatz der TBE im Sinne einer Neugruppierung und Erlösberechnung. Der ermittelte Gesamterlös für das TBE-Verfahren ergab 659.212,19 €. Der Gesamterlös bei Einsatz der TBE liegt damit −7301,94 € unter dem Gesamterlös des Standardverfahrens (666.514,13 €; Tab. 4). Tab. 5 zeigt die TBE-Erlösrechnung im Detail.

**Table 5:** 

Fall #	DRG	VWD	CM	Erlös BFW BW^1^	BWR FP	HOL-Zuschlag	BWR Pflege FP	CM Pflege	Pflegerlös^2^	ZE2023-189^3^	ZE101.01	Summe Erlöse^4^
TBE_1	F42Z	24	6,189	24.895,62 €	6,189	0	1,4478	34,7472	14.113,97 €	15.249,75 €	–	54.259,34 €
TBE_2	F51A	7	4,817	19.376,67 €	4,817	0	1,1676	8,1732	3319,87 €	15.249,75 €	–	37.946,29 €
TBE_3	F42Z	9	6,189	24.895,62 €	6,189	0	1,4478	13,0302	5292,74 €	15.249,75 €	–	45.438,11 €
TBE_4	F51A	10	4,817	19.376,67 €	4,817	0	1,1676	11,676	4742,67 €	15.249,75 €	–	39.369,10 €
TBE_5	F51A	15	4,817	19.376,67 €	4,817	0	1,1676	17,514	7114,01 €	15.249,75 €	–	41.740,43 €
TBE_6	F36B	29	10,619	42.715,56 €	10,619	0	2,7744	80,4576	32.681,07 €	15.249,75 €	–	90.646,39 €
TBE_7	F42Z	31	7,054	28.375,14 €	6,189	0,865	1,4478	44,8818	18.230,54 €	15.249,75 €	–	61.855,43 €
TBE_8	F42Z	32	7,227	29.071,04 €	6,189	1,038	1,4478	46,3296	18.818,62 €	15.249,75 €	–	63.139,41 €
TBE_9	F42Z	12	6,039	24.292,24 €	6,189	0	1,4478	17,3736	7056,98 €	15.249,75 €	–	46.598,97 €
TBE_10	F42Z	19	6,189	24.895,62 €	6,189	0	1,4478	27,5082	11.173,56 €	15.249,75 €	–	51.318,93 €
TBE_11	F51A	26	6,167	24.807,13 €	4,817	1,35	1,1676	30,3576	12.330,95 €	15.249,75 €	42,17 €	52.430,00 €
TBE_12	F51A	5	4,817	19.376,67 €	4,817	0	1,1676	5,838	2371,34 €	15.249,75 €	–	36.997,76 €
TBE_13	F51A	6	4,817	19.376,67 €	4,817	0	1,1676	7,0056	2845,60 €	15.249,75 €	–	37.472,03 €
*Summe*	*–*	*–*	*–*	*320.831,34* *€*	*–*	*–*	*–*	*–*	*140.091,93* *€*	*198.246,75* *€*	*42,17* *€*	*659.212,19* *€*

*#* Nummer, *VWD* Verweildauer, *BWR* Bewertungsrelation, *CM* Case Mix (Summe der Bewertungsrelationen), *BFW* Basisfallwert, *BW* Baden-Württemberg, *FP* Fallpauschale (DRG), *HOL* High Outlier (Langlieger), *ZE* Zusatzentgelt

^1^ Zahl-Landesbasisfallwert BW 2023: 4022,56 €

^2^ Pflegeentgeltwert aus aktuellem Entgelttarif vom 1. März 2023: 406,19 €

^3^ Wert aus Entgelttarif gültig ab 1. März 2023

^4^ Ohne weitere Zu- und Abschläge; basierend auf dem aG-DRG-System 2023

## Diskussion

Vor dem Hintergrund der bevorstehenden Einführung der TBE als thorakaler Seitenarmprothese wurden in der vorliegenden Studie Kosten-Erlös-Analysen anhand von Falldaten des bisherigen Standardverfahren durchgeführt.

Hierin zeigte sich, dass es bei Umstellung der OPS-Codierung auf die TBE zu einer anderen DRG-Gruppierung in den analysierten Fällen (Tab. 4 und 5) kommt. Ebenfalls führt die Verwendung der TBE zu einer Reduktion der Verweildauer und damit Erlösrückgang (u. a. durch Wegfall von Langliegerzuschlägen sowie Anfall von Verlegungsabschlägen; Supplement Tab. 2). Insgesamt reduziert sich der DRG-Erlös bei Verwendung der TBE um ca. 17.000 € (Tab. 4 und 5).

Übergeordnet zeigte sich eine deutliche Abhängigkeit der Gesamterlöse und damit der Wirtschaftlichkeit eines Umstiegs von der ZE-Vergütung. Das ZE2023-189, welches bei der TBE abgerechnet werden kann, führt im Vergleich zu den ZE des Standardverfahrens (ZE2023-50 und ZE105.01) zu ZE-Mehrerlösen von ca. 66.000 €. Mit Blick auf Tab. 4 und 5 kann vermutet werden, dass eine Kostenüberdeckung auch bei Verwendung des TBE wahrscheinlich ist. Hierfür ist jedoch auch die zukünftige Entwicklung des krankenhausindividuell verhandelten ZE20XX-189, welches für 2023 mit 15.249,75 € bewertet ist (OPS 5‑38a.7b), entscheidend. Daten aus der hoch komplexen endovaskulären Versorgung thorakoabdomineller Aortenpathologien zeigen, dass sich diese Eingriffe nicht allein durch den DRG-Erlös abbilden lassen, sondern nur mittels ZE kostendeckend werden können [7]. Hier bedarf es einer intensiven Zusammenarbeit zwischen Klinik und Controlling, um eine belastbare Kostenkalkulation zu erstellen, welche als Basis für ZE-Verhandlungen herangezogen werden kann. Dies ist insbesondere vor dem Hintergrund zu betrachten, dass es sich bei der ZE20XX-189 um ein „Sammelbecken“ für multiple OPS und dahinterstehende SG handelt.

Zu den Kostenaspekten (Supplement Tab. 2) ist zu bemerken, dass die Kostendaten des Standardverfahrens aus unterschiedlichen Behandlungsjahren stammen. Damit unterliegen sie grundsätzlich diversen Verrechnungsfaktoren. Dies kann zu Kostensteigerungen oder Kostensenkungen auf Einzelfallebene führen. In jedem Fall erschwert es die Interpretation der Daten. In einem pragmatischen Ansatz wurden die Kostendaten daher aggregiert. Zwar wurde für die Darstellung die InEK-Matrix verwendet, eine Vergleichbarkeit mit den veröffentlichten Daten der jährlichen Kostenkalkulation liegt jedoch nicht vor. Der Einfluss der TBE auf die Kosten ist schwer abzuschätzen und muss qualitativ bewertet werden. Der (noch unbekannten) Bepreisung der TBE steht der Wegfall der Kosten des Standardverfahrens entgegen. Ebenso reduzieren sich die Kosten für Verweil- bzw. Intensivdauer (Supplement Tab. 1 und 2).

Aus Verfahrenssicht kommt es bei Verwendung der TBE zu einem Umstieg von einem zweizeitigen (Standardverfahren) zu einem einzeitigen Vorgehen. Hierdurch ergibt sich ein Rückgang von sowohl Gesamt- als auch Intensivverweildauer (ca. 5 Tage pro Fall; Supplement Tab. 2). Potenziell werden hierdurch Ressourcen frei, die für weitere Wertschöpfungsprozesse, beispielsweise die Versorgung weiterer Patienten, genutzt werden können.

Perspektivisch steht Deutschland vor einer Krankenhausreform. Alle in dieser Studie enthaltenen Kosten- und Erlösberechnungen basieren auf dem aktuellen System der Betriebskostenfinanzierung von Krankenhäusern in Deutschland. Eine Aussage über die Wirtschaftlichkeit der Nutzung der TBE in einem anderen Vergütungssystem kann daher nicht getroffen werden. Derzeit wird ein Umstieg in ein neues, komplexes System aus fallabhängigen (DRG) und fallmengenunabhängigen Erlöskomponenten („Vorhaltepauschalen für Leistungsgruppen“) diskutiert und geplant.

### Limitationen

Bei der vorliegenden Studie handelt es sich um eine retrospektive Analyse von heterogenen Fällen mit Zone-2-Pathologien. Durch die angewandten Selektionskriterien wurde diese Heterogenität reduziert. Es resultiert jedoch ein hoch selektioniertes Fallkollektiv, was bei der Interpretation der Daten und einem Vergleich mit Mittelwerten umfassenderer Fallkollektive (z. B. DRG-Ebene) beachtet werden muss. Sowohl die Kosten des klinikinternen Standardverfahrens als auch dessen Erlöse werden dabei stark von krankenhausindividuellen Faktoren (u. a. Logistik, Einkauf, jährliche ZE-Verhandlungen) beeinflusst. Ein Vergleich mit anderen operativen/endovaskulären Verfahren (z. B. subklaviale Transposition statt CSCL-Bypass oder Verwendung gebranchter/fenestrierter Bogenprothesen) wurde nicht durchgeführt. Ebenso entfiel eine Betrachtung von Prozess- und Ergebnisqualität. Im Rahmen der Datenaufbereitung wurde jeder Fall auf Plausibilität der Leistungs- und Kostenzuordnung überprüft. Es ist jedoch trotzdem nicht auszuschließen, dass Daten z. T. nicht vollständig erfasst wurden (z. B. bei fehlender Fallzuordnung von Materialien). Die Aggregation der Kostendaten ist als pragmatischer, explorativer Ansatz zu werten. Der überlegene Ansatz der Kostenträgerrechnung war aufgrund der heterogenen Daten aus verschiedenen Jahren nicht sinnvoll. Da klinische Daten zur TBE in der Klinik für Gefäßchirurgie und Endovaskuläre Chirurgie des Universitätsklinikums Heidelberg bisher nicht erhoben wurden, konnte eine Prozesskostenrechnung zum Vergleich der beiden Verfahren nicht durchgeführt werden. Die hier vorgestellte Analyse stellt eine Momentaufnahme bei einem krankenhausindividuellen, hoch selektiven Fallkollektiv dar. Prospektiv-quantitative und/oder verallgemeinernde ökonomische Rückschlüsse sind aus den hier vorgestellten Daten nur sehr eingeschränkt möglich.

## Schlussfolgerung

Vor dem Hintergrund der vorgestellten Kosten-Erlös-Aspekte erscheint bei angemessener Preisgestaltung ein kostendeckender Einsatz der TBE für Zone-2-Pathologien möglich. Prämissen hierfür sind jedoch eine hohe Kodierqualität sowie eine genaue Kosten- und Leistungserfassung. Der zukünftigen ZE-Entwicklung kommt ein entscheidender Faktor für die Wirtschaftlichkeit des Verfahrens zu. Potenziell kann die TBE zu freiwerdenden Operations- und Bettenkapazitätsressourcen führen. Die Auswirkungen der anstehenden Krankenhausreform auf die Wirtschaftlichkeit der vorgestellten Verfahren waren zum Zeitpunkt der Analyse unklar.

### Supplementary Information




