# Supplementary material for: Cost-revenue aspects of endovascular treatment of distal aortic arch pathologies with respect to the introduction of a new thoracic side-branch prosthesis
Source: Chirurgie (Heidelb). 2024 Mar 18;95(6):473–9. [Article in German] doi: 10.1007/s00104-024-02072-3 (PMC11096206; doi:10.1007/s00104-024-02072-3)
Supplement: Supplementary file 1 [file 104_2024_2072_MOESM1_ESM.docx]

**Supplement Tabelle 1: Kostendarstellung für 13 Fälle mit Standardverfahren**

|  | **Personalkosten** | | | **Sachkosten** | | | | | | **Infrastrukturkosten** | |  |
| --- | --- | --- | --- | --- | --- | --- | --- | --- | --- | --- | --- | --- |
|  | Ärztl.  Dienst | Pflege- dienst | med.- techn./ Fkt.-Dienst | Arzneimittel | | Implantate | übriger medizinischer Bedarf | | | medizinisch | nicht-med. |  |
|  |  |  |  | GK | EK | EK | GK | EK | extern |  |  |  |
|  | **1** | **2** | **3** | **4a** | **4b** | **5** | **6a** | **6b** | **6c** | **7** | **8** | **Summe** |
| 01. Normalstation | 1.459,54 € | 2.952,53 € | 1,48 € | 123,46 € | 18,72 € | - € | 201,23 € | - € | 3,10 € | 635,82 € | 1.962,77 € | **7.358,65 €** |
| 02. Intensivstation | 1.551,95 € | 3.426,00 € | 31,19 € | 300,65 € | 24,81 € | - € | 469,74 € | - € | 16,87 € | 481,13 € | 1.401,56 € | **7.703,91 €** |
| 03. Dialyse | - € | - € | - € | - € | - € | - € | - € | - € | - € | - € | - € | **- €** |
| 04. OP-Bereich | 1.457,77 € | - € | 984,24 € | 16,91 € | 7,52 € | 11.438,20 € | 473,93 € | 1.008,05 € | 0,45 € | 546,96 € | 791,15 € | **16.725,17 €** |
| 05. Anästhesie | 734,63 € | - € | 501,21 € | 58,24 € | 5,01 € | - € | 258,36 € | - € | 0,14 € | 160,25 € | 173,90 € | **1.891,74 €** |
| 06. Kreißsaal | - € | - € | - € | - € | - € | - € | - € | - € | - € | - € | - € | **- €** |
| 07. Kardiolog. Diagnostik / Therapie | 27,08 € | - € | 22,56 € | 1,15 € | - € | 20,77 € | 3,80 € | 26,09 € | 0,55 € | 13,46 € | 20,74 € | **136,21 €** |
| 08. Endoskop. Diagnostik / Therapie | - € | - € | - € | - € | - € | - € | - € | - € | - € | - € | - € | **- €** |
| 09. Radiologie | 225,90 € | - € | 146,99 € | 1,22 € | 2,27 € | 29,54 € | 21,19 € | 58,50 € | 0,02 € | 194,16 € | 146,10 € | **825,88 €** |
| 10. Laboratorien | 41,90 € | - € | 198,14 € | 0,01 € | 105,21 € | - € | 244,33 € | - € | 0,03 € | 62,84 € | 137,86 € | **790,32 €** |
| 11. Übrige diagnostische und therapeutisch. Bereiche | 155,08 € | 4,81 € | 308,76 € | 0,37 € | - € | - € | 4,01 € | - € | 0,01 € | 100,16 € | 105,50 € | **678,71 €** |
| **Summe** | **5.653,85 €** | **6.383,35 €** | **2.194,57 €** | **502,01 €** | **163,55 €** | **11.488,51 €** | **1.676,59 €** | **1.092,64 €** | **21,18 €** | **2.194,77 €** | **4.739,58 €** | **36.110,59 €** |

Bei den dargestellten Kostendaten handelt es sich um die gemittelten Werte der 13 Fälle mit Standardverfahren.

EK: Einzelkostenzuordnung; GK: Gemeinkostenverrechnung; Fkt.-Dienst: Funktionsdienst; gelb markiert: Implantatkostenanteil; zur Vergleichbarkeit zwischen den unterschiedlichen Jahren wurden die Daten aus 2018-2022 in die Matrixstruktur von 2017 überführt [nach 10, 11 und 12].

**Supplement Tabelle 2: Verweildauern nach Abschnitten und Eingriffen (in Tagen)**

| **Fall #** | **Gesamt** | | **A**  **–  BP** | **BP**  **–  TEVAR** | **TEVAR**  **– E** | **I  gesamt** | **I**  **vor TEVAR** | **I  BP – TEVAR** | **I  nach TEVAR** |
| --- | --- | --- | --- | --- | --- | --- | --- | --- | --- |
| 1 | 30 | 18,3 | | 6,0 | 6,2 | 11,6 | 11,6 | 0,0 | 0,0 |
| 2 | 10 | 1,1 | | 2,9 | 6,1 | 0,8 | 0,8 | 0,8 | 0,0 |
| 3 | 13 | 0,9 | | 4,0 | 8,5 | 0,0 | 0,0 | 0,0 | 0,0 |
| 4 | 15 | 1,1 | | 5,3 | 9,2 | 0,0 | 0,0 | 0,0 | 0,0 |
| 5 | 21 | 1,2 | | 6,0 | 14,1 | 0,7 | 0,2 | 0,2 | 0,5 |
| 6 | 44 | 16,8 | | 15,0 | 12,0 | 27,0 | 26,1 | 9,3 | 0,9 |
| 7 | 38 | 19,1 | | 7,0 | 12,3 | 20,0 | 15,4 | 1,3 | 4,6 |
| 8 | 35 | 1,0 | | 3,0 | 31,4 | 0,0 | 0,0 | 0,0 | 0,0 |
| 9 | 13 | 3,8 | | 1,0 | 8,3 | 11,9 | 4,7 | 1,0 | 7,2 |
| 10 | 27 | 5,6 | | 8,1 | 12,9 | 1,6 | 0,8 | 0,8 | 0,8 |
| 11 | 34 | 1,2 | | 7,8 | 25,2 | 6,4 | 0,2 | 0,2 | 6,2 |
| 12 | 9 | 1,0 | | 4,0 | 4,2 | 0,0 | 0,0 | 0,0 | 0,0 |
| 13 | 10 | 1,0 | | 4,0 | 5,1 | 0,0 | 0,0 | 0,0 | 0,0 |
| **Mittelwert** | **23** | **5,5** | | **5,7** | **12** | **6,2** | **4,6** | **2,5** | **1,6** |
| **Summe** |  |  | | **73,9** |  | **80** |  | **13,5** |  |

#: Nummer, A: Aufnahme; BP: Bypass; E: Entlassung; I: Intensivaufenthalt; TEVAR: thorakale endovaskuläre Aortenreparatur; **–**: bis.
